# Supplementary material for: Are carbon emissions trading and green financial instruments synergistic? -Comprehensive quantitative research based on content analysis
Source: PLoS One. 2024 Mar 7;19(3):e0298601. doi: 10.1371/journal.pone.0298601 (PMC10919601; doi:10.1371/journal.pone.0298601)
Supplement: S1 Data — (ZIP) [file pone.0298601.s001.zip › Data/地级市Z_F工作报告.zip!/║╙─╧/╔╠╟≡╩╨/└┤╫╘╣1⁄2╓┌║┼í╛┬φ┐╦╩2╛▌═°í┐_2012.docx]

各位代表：

现在，我代表市人民政府，向大会报告政府工作，请予审议，并请各位政协委员和其他列席人员提出意见。

一、过去五年工作回顾

市三届人大一次会议以来，市人民政府在省委、省政府和市委的正确领导下，在市人大、市政协的监督支持下，坚持以科学发展观为统领，团结带领全市人民经历了一系列重大挑战和考验，科学应对、有效处置了强飑线等自然灾害和突发事件，经受了国际金融危机的冲击，主动用领导方式转变加快发展方式转变，圆满完成了市三届人大一次会议确定的目标任务，全市呈现出经济快速发展、事业全面进步、民生持续改善、社会和谐稳定的良好局面。

过去的五年，是商丘又好又快发展的五年。我们坚持发展第一要务，突出产业集聚区、招商引资、重点项目和城镇建设四项重点工作，明确把基础做牢、把总量做大、把产业做优、把持续做好、把保障做强总体要求，擦亮商丘建设、商丘标准、商丘品牌、商丘创造、商丘质量、商丘环境"六大标志"，用领导方式转变加快发展方式转变，保持了国民经济快速健康发展的良好势头。2011年，全市生产总值达到1317．9亿元，年均增长11．9％。地方财政一般预算收入达到56．4亿元，年均增长22．1％。其中，税收收入占财政一般预算收入的比重达到80％以上，是全省税收占比80％以上的3个省辖市之一。全社会固定资产投资完成886亿元，年均增长24．1％。城镇居民人均可支配收入达到16151元，年均增长14．1％；农民人均纯收入达到5637元，年均增长15．5％。产业结构实现了由“二一三”到“二三一”的历史性转变，初步形成了充满活力、富有潜力、支撑有力的现代产业体系。农业基础设施不断完善，农业机械化水平大幅提升，农业综合开发取得显著成效。2011年，粮食总产达121．6亿斤，连年稳定在120亿斤以上，为国家粮食安全作出了突出贡献，被评为“全国粮食生产先进市”。畜牧业持续、快速、健康发展，我市已成为全省重要的优质畜产品生产基地。工业支撑作用日益显现。2011年，规模以上企业达到787家，比2006年增加421家；销售收入超亿元的企业达到270家，比2006年增加212家。规模以上工业实现增加值472．9亿元，年均增长18．9％；实现销售收入1887亿元，年均增长32．9％；实现利润126亿元，年均增长25．7％。现代服务业快速发展，商贸物流、金融、信息、中介等已经成为我市新的经济增长点。2011年，商丘农产品中心批发市场交易额达到150亿元，中央电视台财经频道每天滚动播出的商丘农产品价格信息，已成为全国农产品价格的风向标。

过去的五年，是承接产业转移大见成效的五年。我们坚持实施开放带动战略，积极抢抓产业转移机遇，努力增强发展后劲。围绕主导产业、特色产业，主攻长三角，紧盯闽浙粤，采取“走出去、请进来”的方式，大力开展招商引资活动。发挥区位优势，加强与淮海经济区、陇海兰新经济促进会、大京九经济协作带的战略合作，突出招大引强，加强与央企及国内大企业、大集团的合作，创新招商方式，成功举办了第二、第三届国际华商节、第六届豫商大会等重大节会，招商引资成果丰硕。累计引进省外资金852．3亿元，引进项目2476个，其中引进3亿元以上工业项目68个，投资总额422．3亿元；实际利用境外直接投资5．1亿美元，年均增长17％；出口累计完成4．7亿美元，年均增长20％。我市先后与中石油、国电集团、中建材集团、国药集团等知名企业签署了战略合作协议。飞龙电器、爱玛车业、香雪海电器、万宝制冷集团、国药集团、安踏集团、乔治白、力量新材料集团等一批产业转移项目相继落户商丘。神火集团1�60万千瓦电厂、河南煤化集团千吨级碳纤维、浙江华美河南冰熊制冷公司冷柜、柘城海乐电子、夏邑彩虹纺织、大洋纱线、河南泰丰小鸟电动车等一大批项目竣工投产，为我市经济又好又快发展积蓄了势能，增强了后劲，建设承接产业转移示范区成效明显。

过去的五年，是产业集聚区大发展的五年。我们坚持把产业集聚区作为县域经济发展的重要抓手，作为带动全局的重大战略举措，抓龙头、促配套，抓集聚、促集约，抓创新、促升级，推动产业集聚区实现了大规模、高水平发展。全市11个产业集聚区全部实现了“三规合一”，培育了各具特色的主导产业，产业集聚区已成为转型升级的突破口、招商引资的主平台、农民转移就业的主渠道、改革创新的示范区和县域经济的重要增长极，为商丘又好又快发展提供了有力支撑。全市产业集聚区建成区面积达到86平方公里以上，累计完成固定资产投资1000亿元以上，入驻项目1096个，吸纳就业人员30万人以上。全市制冷产业冰箱冷柜年生产能力将达1000万台，压缩机年生产能力将达1000万套；纺织服装产业上下游链条逐步完善，纺纱能力360万锭，乔治白、阿玛尼、阿迪达斯等国际国内知名服装品牌落户商丘；新材料产业前景广阔，T700宇航级碳纤维填补了中国高端新材料行业的空白，金刚石微粉年产量45亿克拉。2011年，全市产业集聚区实现营业收入1400亿元以上，比上年增长57．5％；实现利润80亿元以上，实现税收35亿元以上，增长76．8％。民权县、柘城县产业集聚区分别进入全省十强、十快行列，有4个产业集聚区纳入省首批示范产业集聚区重点培育范围。

过去的五年，是城乡面貌发生巨大变化的五年。按照“一核两翼组团发展、四位一体统筹推进”的城镇化发展战略，以建设宜居城市为目标，统筹中心城区、县域城区、中心镇区和新型农村社区发展，城镇化水平不断提高。城镇化率达到40．2％，增长11．3个百分点，接近全省平均水平。中心城区以“四城联创”为契机，新修、拓宽、改造了华商大道、神火大道、建设路、中州路等城市主次干道，相继完成了供热、供气、污水垃圾处理、弱电管网、运河治理等一批城建重点工程，中心城区建成区面积超100平方公里，人口达104万，迈入了特大城市行列。商丘新区已经省委、省政府批准，规划建设工作全面启动，17平方公里的商丘新区中心商务功能区规划建设正在积极推进，一批基础设施和产业项目已经开工。我市在全省率先建成林业生态市，先后被授予“中国优秀旅游城市”、“国家园林城市”和“全国绿化模范城市”等荣誉称号。县域城区坚持规划引导、突出重点、突出特色、提高品位的原则，不断完善城市基础设施和公共服务功能，提升了县域城区综合承载能力，永城市跻身全国百强县市，并入围“中部百强”前十强。中心镇区以重点镇、试点镇、特色镇建设为重点，以“30强20快”乡镇创建为抓手，以一镇一品为特色，创建出国家级、省市级重点镇36个，特色乡镇和村庄20个，培育出一批工业型、商贸型、旅游型、特色农业型乡镇。重点围绕产业集聚区、城乡接合部、中心镇区和特色专业村，基本完成了新型农村社区的布点规划，全市新确定的100个社区试点稳步推进。涌现出了永城民生花苑、民权南华社区、宁陵后陈社区、睢县龙王店社区和夏邑冉庄社区等一批规划科学、进度较快、初具规模的新型农村社区典型。

过去的五年，是奠定跨越发展坚实基础的五年。综合交通体系逐步完善，商周高速二期建成通车，连霍高速商丘段改扩建工程、济祁高速公路永城段、210省道豫鲁交界至324省道段二级公路改造项目进展顺利，310国道“二升一”工程、沱浍河航运工程开工建设，商丘机场、郑徐客专商丘场站建设项目开工前的各项工作扎实推进。全市公路通车总里程1788公里，其中高速公路375公里、干线公路1413公里。加强电力基础设施建设，累计投入电网建设资金100亿元以上，投产220千伏及以上输电线路243．8公里，变电容量409．9万千伏安，电力供应得到较好保障。加大水利建设资金投入，实施了中小河流治理、水库除险加固和引黄灌区节水改造等重点工程，17次荣获省“红旗渠精神杯”。实施农村饮水安全工程，完成投资9．05亿元，解决了农村205万人的安全饮水问题。新建了一批广播电视发射台、通讯基站，有线电视、广播覆盖率得到大幅度提升，移动通讯实现了全覆盖。群众生产生活条件得到明显改善。五年经济社会的快速发展，为未来的商丘奠定了良好的基础。今年第一季度，我市经济发展实现了开门红，全市生产总值286亿元，同比增长14．3％，位居全省第二位。规模以上工业增加值完成115．8亿元，同比增长24．7％，位居全省第二位。固定资产投资累计完成140．9亿元，同比增长27．3％，位居全省第三位。公共财政预算收入完成19．3亿元，同比增长34．8％，位居全省第二位。农民人均现金收入1648元，同比增长19％，城镇居民人均可支配收入4894元，同比增长15％。在全省统计的17项主要指标增速中，我市有13项位居全省先进行列，其中有9项位居全省前三位，总体经济运行保持了好的态势、好的趋势、好的气势。

过去的五年，是社会和谐稳定的五年。我们始终坚持改善民生，着力构建和谐商丘。优先发展教育事业，累计投入教育经费202．4亿元，基础教育质量稳步提高，高等教育实现跨越式发展，高校数量达到6所，居全省第4位。我市被国务院确定为“开展地方政府促进高等职业教育发展综合改革试点”城市。实施积极的就业政策，累计新增就业45．4万人。基本建立了覆盖全市城乡居民的养老、医疗、失业、工伤、生育保险体系，城乡居民养老、医疗、工伤和失业保险覆盖率均达90％以上。大力实施保障性安居工程建设，发放廉租住房补贴2．09万户，建设各类保障性住房560多万平方米。文化事业取得长足进步，基础设施不断完善，建成了市体育馆，开工建设了商文化博物馆等。文化产业逐步形成规模，演艺集团、报业集团不断发展壮大，我市被评为“全国文化体制改革工作先进地区”。健全公共卫生服务体系，城乡卫生基础设施建设不断加强。加强人口和计划生育工作，低生育水平连续多年保持稳定。开展社会治安综合治理，深化平安商丘建设，社会治安环境明显改善，群众安全感指数逐年提升，被评为全省平安建设工作先进省辖市。在全省率先启动警务机制改革，公安多项工作在全省领先，“清网行动”七项指标名列全省第一，获公安部贺电表扬。坚持用群众工作统揽信访稳定工作，连续两年实现赴京集访零登记，连续3年被评为信访工作先进省辖市。圆满完成第六、第七届村委会和第三届居委会选举工作。加强土地管理和环境保护，广泛开展节能减排。全面落实安全生产责任制，连续3年被省政府评为安全生产责任目标考核优秀市。加强食品药品安全监管，食品药品安全保障水平持续提高。大力实施科技兴商、质量兴市战略，开展了首届市长质量奖评选活动，科技促进经济发展的作用明显增强，被评为“全国科技进步先进市”。加强公共安全应急管理体系建设，消防、气象、防震减灾等应对各种自然灾害和社会风险的能力不断增强。扎实做好民族宗教工作，巩固了民族团结、宗教和睦、社会和谐的大好局面，被国务院授予“全国民族团结进步模范集体”称号。

过去的五年，是政府工作整体推进的五年。我们坚持依法行政、忠诚履职，深入开展学习实践科学发展观、“两转两提”、“三治三提”推进作风建设专项活动，进一步转变政府职能、转变工作作风，强力推进四项重点工作。在省内率先建成高标准、高质量的行政效能电子监察系统，实现了市、县两级对审批、收费等行为的全程实时监控和管理，提升了行政效能，提升了公务员素质。坚决维护市委权威，认真执行市人大及其常委会的决议和决定，积极支持人民政协履行政治协商、民主监督、参政议政职能，主动听取各民主党派、工商联、无党派人士及人民团体的意见和建议，办理人大代表议案、建议和政协委员提案3815件，满意率在98％以上。顺利完成了市、县两级政府机构改革，精简行政审批事项。推行政务公开和政府信息公开，认真做好市长信箱、公开电话工作，办理来电来信来访10万多次，办结率100％。严格落实党风廉政建设责任制，强化行政监察和审计监督，进一步完善工程招投标、土地招拍挂、政府采购等制度，市政府领导班子向全市公开作出“七不”承诺，塑造了风清气正、廉洁高效的政府形象。

认真开展社会主义法治理念教育，依法治市工作扎实推进。加强社会主义精神文明建设，努力构建社会主义核心价值体系，广泛开展群众性精神文明创建活动，提升了市民整体素质和文明程度，涌现出了商丘水上义务救援队和“贩菜哥”王超华、“弯腰哥”张伟等一批道德模范典型和文明市民标兵，展示了新时代商丘人的新风采、新面貌。全市有5家单位和3个村镇获国家级文明单位（村镇）荣誉称号。工会、共青团、妇联、工商联、社科联等人民团体工作顺利开展。对口援疆工作取得初步成效。

加强国防教育和国防后备力量建设，积极推进军民融合式发展，深入开展双拥共建活动，做好军转安置工作，驻商解放军和武警部队在支持地方建设、抗灾救灾、维护社会稳定方面作出了重要贡献。我市六次蝉联全省和全国“双拥模范城”称号。外事侨务、统计、史志、盐业、海关、检验检疫、工商管理、市场发展、烟草、档案、体育、人防等工作取得新成绩。

各位代表！

回顾过去的五年，我们欣喜地看到，商丘的发展呈现出总体持续、总体提升、总体协调、总体有效的良好态势。今天的商丘，政通人和，充满生机，已站在了新的更高起点上。这一切，离不开省委、省政府和市委的正确领导，离不开市人大和市政协的监督支持，离不开商丘籍和在商丘工作过的老领导、老同志的关心帮助，凝聚着全市各级各部门同心同德、攻坚克难、锐意进取的心血和汗水，铭刻着全市人民与时俱进、干事创业、奋发有为的壮志和豪情。在此，我代表市三届人民政府，向各位人大代表、政协委员，向各位老领导、老同志，向中国人民解放军驻商部队和公安民警、武警消防官兵，向各民主党派、人民团体、工商联和无党派人士，致以崇高的敬意！向所有为商丘建设发展作出贡献的同志们、朋友们，表示衷心的感谢！

成绩来之不易，艰辛铸就辉煌。回顾过去五年的奋斗历程，我们深切体会到：面对纷繁复杂的宏观形势和日益激烈的区域竞争，只有坚持科学发展、积极作为，在形势变化中捕捉机遇，在政策调整中抢占先机，才能有效破解发展难题，增创发展优势，加快发展步伐；面对人民赋予的神圣使命和政府工作的崇高追求，只有坚持市委的正确领导，认真贯彻市委的决策部署，继承发扬政府的优良传统，围绕中心，顾全大局，才能始终做到目标同向、工作同步，不断开创经济社会发展新局面；面对千头万绪的政府工作和急需解决的重大问题，只有坚持重点突破，抓住关键，持之以恒地抓好事关大局的重点工作，才能带动全局，整体推进，取得实实在在的效果；面对人民群众美好期待和财力相对不足的矛盾，只有坚持以人为本、执政为民，千方百计为群众办实事，竭尽全力为群众谋福利，才能团结和调动一切积极因素，凝聚起强大的发展合力；面对赶超发展的艰巨任务和前所未有的困难挑战，只有坚持求真务实、奋发有为，不断创新体制机制，着力提升行政效能，狠抓工作落实，才能迎难而上、开拓进取，把我们的事业不断推向前进！

各位代表！

在充分肯定成绩的同时，我们也清醒地认识到，我市经济社会发展还存在一些困难和问题。主要是：经济总量偏小，人均水平较低，基础差、底子薄的基本市情没有根本改变，与全省及省内兄弟市相比、与周边先进地市相比还有不少的差距；经济结构不优，二、三产占比低的问题依然突出；转型升级任务较重，自主创新能力不强，节能降耗压力较大，经济发展方式仍需进一步转变；政府公共服务和社会管理能力仍需增强，改善民生、普惠于民的任务还比较繁重；政府自身建设还存在不少薄弱环节，庸、懒、散现象在一些职能部门和个别工作人员身上仍然存在等等。对此，我们将高度重视，强化措施，认真解决，努力把各项工作做得更好。

二、今后五年政府工作总体要求和奋斗目标

今后五年，是商丘立足新起点、实现新跨越、再攀新高峰的重要时期，也是加快转变经济发展方式，努力开创“三化协调”、科学发展新局面的关键时期。目前，全市上下思发展、谋发展的氛围日益浓厚，承接产业转移、建设中原经济区、谋划商丘新区等机遇千载难逢。我们要抢抓机遇，科学定位，提升标杆，乘势而上，实现商丘更好更快的发展。

今后五年政府工作的指导思想是：高举中国特色社会主义伟大旗帜，以邓小平理论和“三个代表”重要思想为指导，深入贯彻落实科学发展观，以科学发展为主题，以加快转变经济发展方式为主线，以富民强市为中心任务，按照建设中原经济区、加快中原崛起和河南振兴的总体战略部署，坚持“四个重在”的实践要领，扭住发展不放松，着力扩大内需，着力调整结构，着力改革开放，着力改善民生，全力构筑中原经济区东部战略支撑，努力成为又好又快发展新的经济增长板块，为推进中原经济区建设作出更大贡献。

今后五年政府工作的战略任务是：全力建设“四区两枢纽一基地一中心”，即积极探索并走出一条“两不三新”、“三化协调”科学发展之路，努力建设“三化协调”发展试验区，率先建成中原经济区承接产业转移示范区，着力创建现代农业示范区，精心打造文化体制改革与文化发展先行区，基本建成中原经济区综合交通枢纽和中原经济区物流枢纽，基本形成产业特色明显的新兴工业基地，努力构建豫鲁苏皖四省接合部区域性中心城市。

今后五年政府工作的预期目标是：努力实现“两高一低一提升”，即主要经济指标年均增速高于全省平均水平、高于豫鲁苏皖四省周边地区平均水平，人口自然增长率低于全省平均水平，提升商丘在淮海经济协作区中的地位。

今后五年政府工作的发展愿景是：

——经济发展再上新台阶。经济保持平稳较快发展，粮食综合生产能力不断增强，二三产业比重进一步增加，经济增长质量效益明显提高，人口、资源、环境与经济发展相协调。到2016年全市生产总值翻一番，超过2500亿元，人均生产总值超过2．5万元，公共财政预算收入力争超100亿元，万元生产总值能耗降低16％以上。

——开发开放实现新突破。产业集聚区基础功能完善、综合承载能力增强、产业特色和集聚效应明显。综合交通运输体系基本建成，区位交通优势更加凸显。自主创新体系建设取得重大突破，经济发展环境进一步改善。全方位实施对外开放战略，积极对接长三角和环渤海经济圈，成为承接产业转移的桥头堡；主动融入中原经济区核心增长极，成为中原经济区东部战略支撑，真正实现变边缘为前沿，提升商丘在淮海经济协作区和中原经济区中的地位。开放型经济快速发展，外贸出口和利用外资水平大幅提升，引进市外资金总量突破2000亿元。

——人民生活达到新水平。努力实现居民收入增长和经济发展同步、劳动报酬增长和劳动生产率提高同步，城乡居民收入显著增加，城镇居民年人均可支配收入力争达到2．5万元，农民年人均纯收入超过7500元。社会事业全面进步，教育、卫生、文化等基本公共服务体系不断完善，低收入群体住房条件明显改善，广覆盖、保基本、多层次、可持续的社会保障体系基本建立，社会就业更加充分，人民生活质量明显提高，幸福指数显著提升。

——社会和谐开创新局面。基本公共服务均等化深入推进，民主法制和精神文明建设不断加强，人民群众的利益诉求表达渠道更加畅通。政府公职人员服务意识和能力素养明显增强，群众满意度不断增加。社会管理创新能力进一步提高，应急管理机制更加完善，平安建设扎实推进，群众安全感显著提升，基层化解社会矛盾的体制机制更加健全，社会大局和谐稳定，平安建设工作持续保持在全省的先进位次。

——城乡建设展现新面貌。新型城镇化引领作用不断提升，城乡一体化进程明显加快，城镇化率力争超过50％。中心城区建成区面积125平方公里以上，总人口超过120万人。城乡基础设施更加完善，城镇规划建设和管理水平显著提高。商丘新区建设初具规模，县域城区和中心镇区更具特色。新型农村社区建设加快推进，现代城镇体系和现代产业体系基本建成。“三商”特色文化的影响力和竞争力日益增强，城乡文明程度和市民文明素质显著提高。

——生态环境得到新提升。资源节约型、环境友好型社会建设加快推进，人与自然和谐相处。环境综合整治成效明显，能源结构更加优化，传统能源清洁高效利用，循环经济发展水平进一步提升，节能减排目标圆满实现。水资源保护利用能力不断增强，人民群众饮水安全得到有效保障。人居质量明显改善，努力实现天蓝、地绿、水清、景美的美好愿望，“长寿之乡”美誉度进一步提升。

我们相信，经过五年的努力，一个经济繁荣、实力雄厚的新商丘，一个形象靓丽、环境优美的新商丘，一个社会和谐、人民富裕的新商丘，必将展示在世人面前，屹立在豫东大地！

三、2012年工作任务

今年是实施“十二五”规划承上启下的重要一年，也是全面贯彻省九次党代会精神和推进中原经济区建设的重要一年。做好今年的政府工作，对于落实市四次党代会精神，巩固经济社会发展良好势头，全力构筑中原经济区东部战略支撑，实现全市经济社会又好又快发展具有十分重要的意义。

根据市委经济工作会议确定的总体要求，今年经济社会发展的主要预期目标是：全市生产总值增速高于全省平均水平；公共财政预算收入增速高于生产总值增速2个百分点；全社会固定资产投资增长22％以上；社会消费品零售总额增长17％；外贸出口增长21％；实际利用外商直接投资增长20％；城镇居民人均可支配收入和农民人均纯收入分别增长9％、10％；人口自然增长率控制在6．5％。以内；居民消费价格涨幅4％左右；节能减排完成省定目标任务。

围绕以上目标任务，重点抓好以下十个方面的工作：

（一）以商丘新区为龙头、新型农村社区为重点，构建新型城镇化体系。围绕“一核两翼组团发展、四位一体统筹推进”的城镇化发展战略，认真实施城乡建设三年行动计划，全面落实各项目标任务。一是加快商丘新区建设。完成商丘新区总体规划，启动重点开发区域控制性详细规划和专项规划。搞好新区交通设施建设，构建骨干交通体系，升级改造310国道、105国道新区路段，开工建设105国道高架桥。加快商虞一体化进程，以道路互联互通为基础，实现规划、功能一体化。推进豫东综合物流集聚区基础设施建设，启动环保设备工业园区规划和基础设施建设。全力打造新区产业体系，积极发展现代制造业、新材料工业、现代服务业、现代物流业，做大做强城区经济。重点抓好17平方公里商丘新区中心商务功能区建设，搞好睢阳大道、宋城路、中州路3条主干道和与之相配套的6条次干道等基础设施建设，初步形成支撑商丘新区中心商务功能区建设的路网框架。以“四城创建”为载体，加快中心城区建设，强化城市精细化管理。以内河水系治理、道路升级改造、公园绿地建设为重点，围绕建设宜业宜居宜游特色魅力城市，抓紧实施城市道路、市容环卫、公交、供水、排水、燃气、集中供热、城市照明等专项规划。商文化博物馆建成投入使用，开工建设老干部活动中心。按照组团式发展的理念，抓好中心商务功能区和特色商业街示范创建。二是加快新型农村社区建设。充分发挥新型农村社区在新型城镇化中的战略基点作用，突出规划先行，把新型农村社区建设纳入城镇体系规划，年底前基本完成全市新型农村社区建设详规。市、县两级财政投入资金2亿元，整合涉农项目资金3亿元以上，引导社会资金参与新型农村社区建设。今年新开工建设100个新型农村社区试点，基本完成产业集聚区内新型农村社区建设任务。三是加快县域城区建设。充分考虑区位、资源、人文、产业等特点，科学定位县城发展，坚持高起点、高标准推进县城规划建设，实施联动组团式发展。加大旧城、城中村开发改造力度，选择有代表性的项目作为试点，以点带面，形成各具特色的县域城区。四是加快中心镇区建设。把重点城镇发展作为统筹城乡发展的战略节点，促进城市基础设施向农村延伸，城市公共服务向农村覆盖，城市文明向农村辐射。加强中心镇与专业园区、新型农村社区建设的衔接，推进产业融合发展与农民就近就业转移，培育特色乡镇。

（二）以做大总量为基础，打造新兴工业基地。按照“特色主导、龙头带动、创新驱动、集约高效”的原则，发展壮大主导产业，改造提升传统产业，积极培育新兴产业，构建现代产业体系。全市规模以上工业增加值同比增长16％左右，实现主营业务收入2100亿元以上，实现利税总额200亿元以上，主营业务收入超亿元的企业达300家左右。一是发展特色主导产业。着力发展食品、纺织服装、装备制造、制冷四大特色主导产业，实现主营业务收入1000亿元以上。积极开发功能营养食品、高档服装、电动车、冷藏保温车等中高端产品。打造“一谷两城两基地”，即“中原冷谷”、“全国新型食品工业城”、“中原经济区纺织服装城”、“全国环保专用设备生产基地”、“全国电动车生产基地”。二是提升传统优势产业。改造提升煤炭、有色、化工等传统产业，积极开发煤制烯烃、铝型材、铝板带箔、高档汽车轮毂等精深加工产品。建设“中原经济区铝精深加工基地”、“中原经济区煤化工基地”。传统产业主营业务收入力争超过800亿元。三是培育战略新兴产业。重点发展高性能纤维产品和复合材料、金刚石微粉及其制品、生物医药、集成电路、电子元件等产品，逐步提升新兴产业产值比重和发展权重。建设“全国碳纤维生产研发基地”、“全国超硬材料生产基地”。新兴产业实现主营业务收入100亿元以上。四是壮大龙头骨干企业。培育扶持60户规模效益企业和60户高成长型企业，提升永煤集团、神火集团、科迪集团等大型企业集团的辐射带动能力。加快中小企业技术进步和结构调整，着力培育发展一批“专、精、特、新”型中小企业。五是优化产业布局。加快中心城区工业经济发展，充分发挥城区经济对区域经济的辐射带动作用。加快推进虞城县科迪食品制造业基地、开发区机电装备（电动车）产业基地、民权县制冷产业基地、永城市煤化工产业基地四个省级新型工业化产业示范基地再上新台阶。

（三）以产业集聚区建设为平台，培育区域经济增长极。按照提升速度、提高水平、扩大效果的要求，着力补齐短板、破解制约、提高集聚、提高质量、提高效益。一是培育产业集群。围绕主导产业，明确招商对象，促进产业配套，延伸产业链条，突出龙头带动，统筹推动同类和关联项目按照主导产业集中布局建设，促进集群发展，培育一批超50亿元、超100亿元的产业集群，持续提升产业集聚发展水平。二是完善服务功能。提升集约化发展和要素保障水平，重点抓好资金融通、土地整理、人才和人力资源、技术创新孵化“四个平台”建设。不断加强基础设施、生产和生活服务配套能力建设，形成更强竞争力。三是搞好示范创建。对列入省重点培育示范产业集聚区范围的4个产业集聚区，分别对照标准，完善功能，提升档次，争取早日通过省直有关部门的认定。未列入省重点培育范围的，明确创建目标和规划，制定创建工作保障措施，争取早日列入省重点培育产业集聚区范围。四是注重产城融合。在完善产业集聚区发展规划的基础上，高标准、高起点做好空间规划和控制性详细规划编制。促进城镇基础设施向产业集聚区延伸，城镇现有设施与产业集聚区衔接共享，实现以产带城、以城促产、产城一体化。

（四）以实现农民增收为目标，加快农业现代化进程。坚持把“三农”工作放在重要位置，努力在粮食增产、农业增效、农民增收上下工夫。一是稳定粮食综合生产能力。抓住我省实施高标准良田“百千万”工程的机遇，规划建设一批百亩方、千亩方、万亩方高标准粮田，切实做好中低产田改造、高标准农田示范工程建设，大力推广先进适用技术，积极开展高产创建，确保今年粮食综合生产能力稳定在120亿斤以上。二是促进农村经济结构调整。大力发展现代畜牧业，加快标准化规模养殖和畜牧产业化集群建设，推进标准化生产和产业化经营，提高特色、优质畜产品比重。积极发展林果、蔬菜、花卉等高效农业，加快实现从农业大市向农业强市转变。三是加快农业产业化集群发展。立足农业资源优势，培育一批产值超亿元的农业产业化重点龙头企业。以龙头企业为支撑，重点抓好乳品、面品、肉品产业化集群建设，提高农业产业化经营质量和效益。加快推进农业科技创新，持续增强农产品供给保障能力。加快发展农民专业合作组织，为农业产业化集群发展提供有效的服务。四是加强农业基础设施建设。紧紧抓住中央加大对农田水利基本建设投入的机遇，重点抓好三义寨引黄灌区续建配套节水改造、任庄水库除险加固等一批农田水利基本建设项目。加快推进农业机械化，为新型农业现代化提供装备支撑。加强农村基础设施建设，重点抓好农村道路、农村人口饮水安全工程建设。加大扶贫开发力度，实施整村推进工程。推进农村环境治理，抓好村镇绿化美化，持续改善农村生产生活条件。加快实施豫鲁苏皖人工影响天气跨区作业基地项目前期工作。五是拓宽农民增收渠道。认真落实各项强农惠农政策，增加农民政策性收入。鼓励农民优化种养结构，发展立体种植、规模养殖和深度加工，增加农民经营性收入。大力开展“阳光工程”、“雨露计划”和农民专业技能培训，加快实施农民创业工程，提高农民工资性收入。大力推进农民以转包、出租、互换、转让、股份合作等形式流转土地承包经营权，分享土地承包经营权流转中的增值收益，允许其通过市场自愿转让获得财产性收入。

（五）以重点项目建设为引擎，进一步增强经济发展后劲。深入开展“项目突破年”活动，努力提高重点项目在固定资产投资额中的比重，用重点项目的发展促进主导产业集聚。一是积极谋划储备。抓住中原经济区建设、产业转移及国家支持“三农”、交通、基础设施建设和社会事业发展的重大机遇，结合我市实际，精心谋划，筛选、编报一批建设项目，并搞好项目对接，为项目的落地打下坚实基础。二是加快建设进度。按照“有限资源保重点、集中力量办大事”的原则，着力保障重点项目建设的要素供给，确保重点项目建设施工和生产的有效推进。开工建设商丘机场、神火集团2�35万千瓦热电联产机组，抓好永煤集团顺和煤矿、连霍高速拓宽改造，强力推进中石油商丘千万吨炼油厂、国电民权二期等重大项目前期工作。三是增强服务保障。继续实施项目谋划、联审联批、市领导联系重大项目、项目建设进度推进和考核奖惩“五项机制”，进一步优化建设环境，严禁对重点项目乱检查、乱评比、乱收费、乱摊派，严厉打击一切阻碍项目建设的不法行为。加强对重点项目的监督检查，确保建设进度，确保资金安全和施工安全，确保工程质量。

（六）以实施开放带动为主战略，建设承接产业转移示范区。抢抓承接产业转移的机遇，着力打造内陆开放高地。一是增强开放意识。全市各级各部门领导干部，要把扩大开放作为“一举应多变”、“一招求多效”的有效抓手，树立对外开放“一盘棋”思想，突出整体作战，着力构建“举市开放”体制，形成开放发展的强大合力。二是加大招商引资力度。积极“走出去、请进来”，围绕主导产业招商引资，积极承接产业转移，抓好延链和补链招商，以产业链整合提升为重点，提升关键环节，完善配套能力，提高市场竞争力。对铝深加工、家电制冷、电动车及零部件、新材料、纺织服装等重点产业，要在延链、补链上率先取得突破。大力推进产业集聚招商，按照“大项目—产业链—产业集群—产业基地”的思路，承接境外和沿海发达地区链式和集群式产业转移，争创一批国家和省级对外开放示范集聚区，推进商丘经济开发区申建国家级开发区。三是打造招商引资平台。认真组织参与中博会、厦洽会和豫港澳、豫台、豫沪等经贸洽谈活动，组织好我市自主举办的“5＋2”经济合作等重点招商活动，加大项目推介力度，积极拓展与世界500强、国内500强战略合作的广度和深度，努力实现引进市外资金500亿元以上的目标，力争再引进一批科技含量高、投资规模大、带动能力强的重大项目。四是优化对外开放环境。建立招商引资受理、办理联席会议制度，健全招商引资和项目落地衔接机制，全面推行首问负责制、服务承诺制、限时办结制、无偿代理制，进一步简化审批程序，提高审批效率，增强审批透明度，完善一站式、一条龙服务，倾力打造“低成本、高效率、无干扰”的开放发展环境。五是健全激励机制。实行招商引资“一把手”负责制，对重点活动、重大项目，主要领导亲自指挥，亲自参与洽谈和督查落实。实行市领导巡回检查招商引资重点项目制度、招商引资“月统计、月备案、月督查、月通报”制度，继续实行奖惩制度，充分调动全市干部群众招商引资的积极性和主动性。

（七）以构建综合交通体系为契机，推进中原经济区综合交通枢纽和物流枢纽建设。围绕巩固提升区位交通优势，加强现代服务业体系建设的任务，一是持续完善综合交通体系。完成连霍高速商丘段改扩建工程、310国道“二升一”改造工程，济祁高速连霍至永登段建成通车，抓好沱浍河航运工程建设，尽快开工建设商丘机场、郑徐高铁客运专线及商丘火车站“三站合一”工程，争取开工建设商登高速公路项目和105国道“二升一”改造工程。加快培育集铁路、公路、水运、航空为一体的中原经济区综合交通运输枢纽，进一步凸显商丘在中原经济区中的交通枢纽地位。二是突出发展商贸物流业。加快豫东综合物流集聚区建设进度，重点做好亿丰国际商业博览城、国家现代农产品流通试点、白云物流仓储等项目，逐步谋划纺织服装批发市场、超硬材料专业市场、制冷产品及设备专业市场和物流园区建设。加快物流专业市场建设，改造升级商丘农产品批发市场、中原车城，繁荣光彩大市场、中意建材等专业市场。规范发展小商品批发市场、城区农贸市场和乡镇集贸市场，着力提高市场的覆盖水平。三是积极拓展金融服务业。大力发展现代金融业，积极引进股份制银行，支持国内外金融机构在我市设立银行、证券、保险、信托、金融租赁、期货经纪等金融机构及分支机构。积极推动银企对接，着力扩大融资规模。加快发展村镇银行，规范担保业，为“三农”和中小企业融资提供支持。四是加快发展新兴服务业。大力发展信息服务业，做好无线城市规划和建设，推动基础通信网络、互联网、数字电视网的“三网”融合，推动网络信息产业升级。以市场为导向，加强社区服务设施和网点建设，重点发展家政服务、养老服务等业态，健全完善社区服务体系。五是大力发展旅游业。按照“豫东平原文化旅游”的定位，努力打造商丘古文化旅游区和豫东旅游集散中心，大力发展商丘文化游、生态游、红色游、城市游、乡村游和特色游，提升商丘古城、芒砀山、黄河故道等精品景区品位。

（八）以深化改革为动力，着力创新体制机制。坚定不移地深化重点领域和关键环节改革，加快构建有利于构筑中原经济区东部战略支撑的体制机制。一是破解瓶颈制约。完善土地保障机制，大力推进土地综合整治，推进城乡建设用地增减挂钩，节余指标优先用于产业集聚区发展。对存量建设用地清理整治，促进土地集约节约利用。完善资金保障机制，深化投融资体制改革，完善金融体系建设，做强做实政府投融资平台，积极引进战略合作者，吸引有实力的大企业、大集团参与投融资平台建设。大力支持科迪集团、中分仪器、张弓酒业等企业上市融资。加快城投债发行步伐，鼓励有条件的企业扩大直接融资规模。完善人力资源保障机制，深入实施人才发展规划，统筹推进各类人才队伍建设，充分发挥商丘职业院校和职业技能培训机构的作用，结合产业集聚区主导产业用工需求，实行订单式、一对一培训，为承接产业转移提供人才支撑。二是创新社会管理。推进平安商丘建设，健全人防、物防、技防相结合的治安防控体系，严厉打击各种刑事犯罪，确保人民群众生命财产安全。加强重大项目、重大决策的社会稳定风险评估，健全领导干部接访、下访、回访和包案长效机制，深入排查化解矛盾纠纷，集中解决信访积案和突出问题，切实维护群众合法权益。实施“六五”普法规划，进一步加强司法救助和法律援助。加强政府诚信、商务诚信和司法诚信建设，完善社会信用体系。加快公共应急体系建设，预防和妥善处置各类突发事件，提高公共安全保障能力。三是加快科技创新。积极发展各类企业研发中心、科技孵化中心等创新载体，完善产学研合作机制和科技成果转化机制，加速科技成果的转化应用。加快建设“河南省制冷专业院士工作站”、“碳纤维新材料研发中心”、“电解铝节能研发中心”和“国家级钢卷尺及五金工具产品质量监督检验中心”，发挥“中国制冷机电产品质量检测检验中心河南站”、“河南省金刚石微粉检测站”作用，提升解决产业发展关键技术问题的能力。引导有条件的产业集聚区创建省级高新技术产业开发区，膨胀高新技术产业规模。四是鼓励自主创业。进一步培育各类创业主体，实施积极的创业政策。培养和引进创业人才，逐步壮大懂经营、善管理的创业队伍。坚持平等保护物权，营造全社会支持创业、鼓励创业的良好氛围。五是推进重点领域和关键环节的改革。深入推进国有企业改革。支持非公有制经济和中小企业发展，加快中小企业特别是微型企业服务体系建设。继续深化医药卫生体制改革，全面提升食品药品安全水平。积极开展文化改革发展试验区综合配套改革，推动文化产业向纵深发展。推进行政管理体制改革，清理调整行政审批事项，推行政府绩效管理和行政问责制度，扩大政务公开范围和层次。

（九）以让人民群众得到更多实惠为目的，持续推进和谐商丘建设。一是稳定和扩大就业。坚持就业优先战略，继续实施更加积极的就业政策。重点扶持就业容量大的现代服务业、创新型科技企业和小型微型企业，创造更多就业岗位。加强退役军人技能培训与就业安置工作。实施小老板创业计划，以创业促就业。二是加快完善社会保障体系。加快完善覆盖城乡的社会保障体系。基本实现新农保和城镇居民社会养老保险制度全覆盖。切实解决失地农民生活保障问题。大力发展社会福利和慈善事业、妇女儿童事业、老龄事业、残疾人事业。三是强力推进保障性安居工程建设。坚持分类指导，科学规划，优化结构，确保质量。多渠道筹集保障房源，在商品房项目中配建一定比例的保障房，同步建设和完善必要的市政配套服务设施。四是优先发展教育事业。实施学前教育三年行动计划，加大公办幼儿园建设力度。优化中小学校布局，加快推进义务教育学校标准化建设，完善中小学教师支教交流制度，推进义务教育均衡发展。制定切实可行的政策措施，推进民办教育健康发展。五是积极发展医疗卫生事业。加强城市社区卫生服务，完善农村基层医疗卫生服务网络，提升城乡医疗服务能力。加强市、县急救指挥中心基础设施建设，建立完善医疗急救体系。加强重大传染病防控体系和卫生监督体系建设。建立城乡居民大病医疗救助制度，提高救助标准。实施基本药物制度，加强乡村医生队伍建设，清理化解基层医疗卫生机构债务，年底前全面完成村卫生室标准化建设。

六是统筹做好人口和计划生育工作。坚持和完善现行生育政策，稳定低生育水平，提高出生人口素质，关爱计划生育家庭。加强流动人口管理和服务，营造和谐人口环境，促进家庭文明幸福。七是持续推进文化名市建设。坚持早谋划、早运作，抓重点、抓细节，把2012年中国�商丘国际华商节办成一个高品位、有特色的经济文化盛会。认真办好河南省第五届黄河戏剧节。扶持商丘演艺集团做大做强，完成市县文化院团转企改制。加快文化产业园区和文化产业示范基地建设，形成文化产业集聚效应，促进文化产业规模化、集约化和产业化发展。加快文化基础设施建设，继续完善乡镇文化站、农村书屋等文化惠民工程。进一步繁荣文化创作，切实加强文化遗产、非物质文化遗产的宣传、开发和保护。继续做好大运河申遗和古城保护工作。大力发展体育事业，不断提高全民健康素质，办好市第四届运动会。八是持续加强安全生产工作。全面落实安全生产责任制，扎实开展安全商丘创建活动，建立健全安全生产支撑保障体系，保持安全生产形势持续稳定。九是切实抓好食品药品安全管理。大力推进食品药品安全示范县创建工作，加强市场监管，保障人民群众健康安全。十是持续办好事关民生的十件实事。

加强国防教育和国防后备力量建设，支持驻商解放军、武警部队、预备役部队、民兵和人民防空建设，促进军民融合式发展。深入开展双拥共建，巩固发展军政军民团结。抓好民族和谐教育，依法依规管理民族宗教事务，加快少数民族地方经济社会发展。支持工会、共青团、妇联等人民团体发挥桥梁纽带作用。切实做好外事侨务、对台工作。认真做好统计、史志、气象、地震、社科研究等工作。继续做好对口援疆工作。

（十）以解决人民群众切身利益问题为出发点，继续办好十件实事。一是千方百计扩大就业。实施全民技能振兴工程，全年完成各类培训11．5万人。开展好成人职业培训工作，完成中专招生3万人。全市新增城镇就业人口5．6万人；新增农村劳动力转移就业9万人；政府购买公益性岗位6000个，帮助“4050”人员及零就业家庭人员在内的就业困难群体就业；切实做好高校毕业生、城镇就业困难群体和残疾人就业帮扶工作，全年完成帮扶就业1．1万人。二是提高社会保障水平。企业退休人员在去年月人均养老金1345元的基础上，今年人均提高10％以上；新型农村合作医疗和城镇居民基本医疗保险财政补贴标准，由人均200元提高到240元；建立社会救助和保障标准与物价上涨挂钩联动机制；开展城乡医疗救助“一站式”结算服务；全面建立农民工工资支付保障制度，加快推进工资支付保证金制度和工资支付监控制度，确保农民工工资发放。三是推进保障性安居工程建设。新建廉租房12540套、公共租赁住房5600套、经济适用住房1520套，城市和国有工矿棚户区改造2876户，林业棚户区改造704户，垦区棚户区改造760户。四是关心救助弱势群体。深入开展法律援助工作，在去年受理案件2600件、接待咨询13000人次的基础上，努力扩大免费法律援助范围，确保应援尽援。实施残疾人康复工程，为4万名残疾人提供康复服务。完成10．37万农村贫困人口的脱贫任务。五是搞好医疗卫生保障。全市免疫规划人数达到350万人次，接种率达到95％以上；实施基本药物制度，加强乡村医生队伍建设，清理化解基层医疗卫生机构债务，结合新型农村社区建设，基本完成已建成的新型农村社区卫生室标准化建设任务。六是改善学校办学条件。改造市一中、行知学校校舍危房16000平方米，完成商丘中专数控技术实训楼和幼师新校区、医专新校区、一高新校区一期工程建设；完善市特殊教育学校和职教中心基础设施；完成柘城、民权、宁陵三县中小学生营养餐供应试点工程；完成全市新型农村社区中小学校、幼儿园规划布局方案。七是改善农村生产生活条件。开工建设310国道135公里一级公路主体工程建设。筹措资金1．5亿元，新改建农村公路200公里。筹措资金2．32亿元，解决50万农村人口安全饮水问题。开工建设100个新型农村社区。八是优化城市居民居住环境。全市完成旧城、城中村拆迁面积360万平方米，新建房屋360万平方米。投资约2亿元，继续实施城区内河水系综合治理工程，实施康林河、忠民河污水管网铺设和南京路泵站建设工程及包河（民主路～南京路）等河道清淤、砼挡墙、绿化、硬化、亮化等景观节点建设工程。实施引黄调蓄工程，健全城区水源水系。完成文化路、凯旋路、归德路、南京路四条道路示范段工程建设任务，提升城市核心区综合品位。九是改善城区交通状况。购买新型空调公交车辆，调整增加公交线路，改善居民乘车条件；打通断头路，消除“肠梗阻”，整修紫荆路（南京路～北海路）、八一中路（新建路～高炮旅）、货场东路（长征路～新建路）、天瑞路（民主路～陇海路）等道路，改善路况路貌；对归德路南段和华商大道东段升级改造。开工建设郑庄枢纽立交桥和平台枢纽立交桥工程。十是增强公共服务功能。强力推进商丘新区中心商务功能区建设，完成中心商务功能区城市设计、水系规划、基本路网建设，启动征地拆迁和安置房建设工程、高端城市综合体建设，开工建设豫东大剧院、明月湖、日月塔、信华五星级国际酒店等一批城市标志性建筑；发行“商丘便民卡”，为居民提供安全、便捷的金融支付服务；投资4亿元，升级改造商丘农产品中心批发市场，提升市场档次；开工建设和升级改造城乡农贸市场50个，解决农产品市场脏乱差、占道经营等问题。

四、切实加强政府自身建设

做好新一届政府工作，必须坚持以领导方式转变加快发展方式转变，认真落实“三具两基一抓手”，深入推进“两转两提”，巩固扩大“三治三提”活动成果，促使全市政府公职人员勤政为民、锐意进取的良好风气深度养成、普遍形成，内化于心、外践于行，持续提升政府公信力和执行力。

（一）坚持执政为民。坚持以人为本，牢记发展为民，始终把人民利益放在第一位，把工作出发点和落脚点放在为商丘人民谋利益、谋福祉上。加大对改善民生的投入力度，构筑社会保障体系，提高保障补助标准，拓宽群众增收渠道，改善生产生活条件，切实解决老百姓住房、看病、优质教育资源共享等问题，让人民群众更多地分享发展成果。深入治理征地拆迁、住房保障、食品药品安全、环境保护、安全生产等方面的突出问题，切实维护人民群众合法权益。

（二）坚持务实发展。强化务实发展意识，大兴求真务实之风，察实情、鼓实劲、出实招，反对急功近利、华而不实的形象工程、政绩工程，自觉做到不动摇、不懈怠、不折腾。注重真抓实干，少提口号，少讲空话，说到做到，说好做好，将战略谋划和决策部署真正落实到一个个具体项目上，落实到一个个责任主体上。进一步树立务实、勤奋、高效的良好形象，努力打造服务型政府。

（三）坚持依法行政。加强法制教育，规范行政行为，建设法治政府，做到依法行使职责。严格执行人大及其常委会的决议决定，自觉接受人大法律监督和政协民主监督，认真办理人大代表和政协委员的议案、提案和建议。带头遵守法律规章，健全科学民主决策制度，坚持重大事项集体研究决定。严格落实行政执法责任追究制，强化行政执法监督，做到规范执法、文明执法。加大政务公开和政府信息公开力度，畅通政府与公众互动渠道，确保行政权力在阳光下运行。

（四）坚持廉洁从政。落实党风廉政建设责任制，扎实推进惩治和预防腐败体系建设，加强廉政文化建设，加大纠风和专项治理力度，着力解决人民群众反映强烈的突出问题。严格执行领导干部廉洁从政各项规定，有效预防各种腐败行为的发生。加强廉政监督检查，针对违纪违法行为易发多发领域和关键环节，规范办事程序，完善规章制度，做到治标与治本、惩治与预防同步推进。坚决反对铺张浪费，严格控制和压缩“三公”消费，推进公务消费制度改革，努力建设节约型机关。

各位代表！

回顾过去，取得的成绩令人鼓舞；展望未来，发展的蓝图催人奋进。在全面推进中原经济区建设的大潮中，我们一定牢记使命，坚定信心，务实进取，开拓创新，全力构筑中原经济区东部战略支撑，为实现商丘经济社会又好又快发展作出新的更大的贡献，以更加优异的成绩迎接党的十八大胜利召开！
